# Twiddler Syndrome without Lead Dislodgment Discovered by Remote Monitoring

**DOI:** 10.1155/2021/8816524

**Published:** 2021-02-06

**Authors:** Catherine Champagne, Nicolas Dognin, Franck Molin, Christian Steinberg, François Philippon, Jean-François Sarrazin, Gilles O'hara, Isabelle Nault, Karine Roy, Benoit Plourde, Louis Blier, Jean Champagne

**Affiliations:** Department of Cardiology, Institut Universitaire de Cardiologie et de Pneumologie de Québec, Laval University, Quebec, Canada

## Abstract

Twiddler syndrome is an uncommon yet dangerous phenomenon usually resulting in lead displacement with loss of capture of cardiac implantable electronic devices. In this case report, we present an interesting case of Twiddler syndrome without lead dislodgment which was detected by an alert triggered by an increase in impedance on remote monitoring.

## 1. Introduction

Twiddler syndrome is a rare complication (0.07-1.1%) following the implantation of cardiac devices and has been observed with different types of cardiac devices [1–4]. Twiddler syndrome is characterized by repeat, patient-initiated manipulation of the pulse generator resulting in rotation of the device and lead twisting that may result in dislodgment of the electrodes and loss of capture. Here, we report a rare case of Twiddler syndrome that was diagnosed with the help of remote monitoring.

## 2. Case Presentation

A 75-year-old woman with a mixed ischemic/nonischemic cardiomyopathy and depressed left ventricular systolic function (ejection fraction of 25%) underwent the implantation of a single-chamber primary prevention ICD (Boston Scientific Autogen) in January 2017. Her past medical history was significant for multiple other cardiovascular comorbidities including stage III obesity with a body mass index of 41 kg/m^2^. She was not known for a history of dementia or impaired cognition.

**Figure 1:**
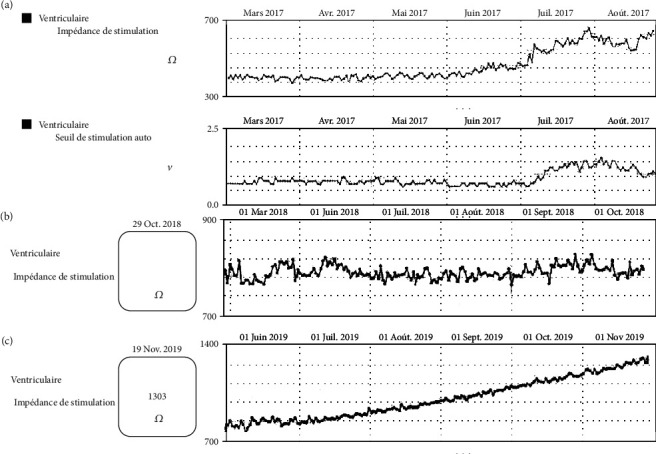
Impedance and pacing threshold: (a) simultaneous elevated impedance and threshold six months postimplantation; (b) stabilized impedance and threshold in 2018; (c) remote monitoring of the RV lead showing evidence of an increased impedance.

**Figure 2:**
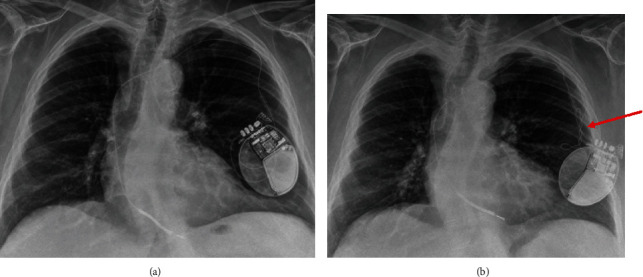
PA chest X-ray one month postimplantation (a) and 3 years postimplantation showing twisting of the lead (b) (red arrow).

**Figure 3:**
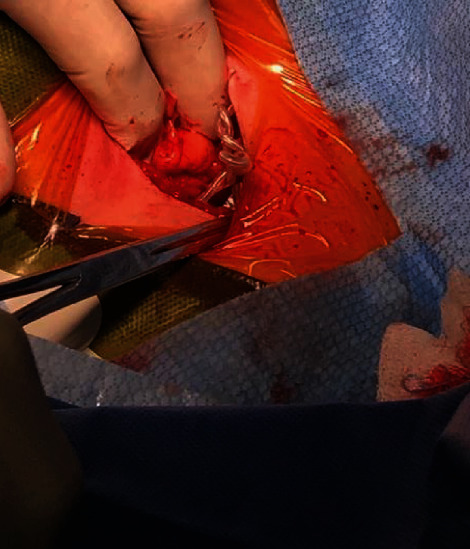
Lead wrapping observed during surgical revision.

An active fixation right ventricular (RV) lead (Boston Scientific Reliance 4-Front DF4) was implanted at the RV apex. Six months postimplantation, the RV lead pacing threshold and impedance increased from 0.7 V to 2 V and from 450 ohms to 800 ohms, respectively (Figure 1(a)). It was still considered clinically acceptable and then remained stable for 2 years (Figure 1(b)). No chest X-ray was done during that period. In November 2019, a remote monitoring alert was triggered due to a high pacing impedance. A hospital visit was scheduled to verify the patient's ICD. The patient was asymptomatic and she denied any manipulation of device. The pacing impedance was increased to 1400 ohms, whereas the shock impedance remained stable at 90 ohms (Figure 1(c)). The RV pacing threshold was also increased to 2.9 V at 0.8 msec. Sensing remained unaffected showing a detection of intrinsic R-waves of 10 mV. No noise or inappropriate ventricular arrhythmia therapy had been recorded by the device. A PA/lateral chest X-ray showed typical signs of a Twiddler syndrome (Figure 2), and the ICD lead showed a reduced lead slack with the tip at the RV apex. The patient was admitted for surgical revision.  Figure 3 shows the intraoperative device situation after opening of the pocket capsule. The suture sleeve was intact and still tight. The new electrode was placed one centimeter above the old abandoned one. It was impossible to insert a stylet inside the defective ICD lead and an extraction was not performed. The pulse generator was not changed as its longevity was still predicted to last 10 years, but it was fixed to the *pectoralis major* in a new subcutaneous pocket with a suture in the header of the device to prevent any migration.

## 3. Discussion

This case report illustrates a Twiddler syndrome without lead dislodgment detected by an impedance lead alert from the remote monitoring system. Failure of the device is often the clinical manifestation of Twiddler syndrome [1], and this may have catastrophic consequences in pacemaker-dependent patients. In patients with ICD, it often presents with the inability of the device to properly shock ventricular arrhythmias and inappropriate ICD therapies and sometimes may lead to sudden cardiac death [5]. The only sign of Twiddler syndrome in this patient was the constant increase in pacing threshold and pacing impedance. Contrarily to most Twiddler syndromes that have been previously reported, the electrode was still tightly attached to the suture sleeve and in contact with the right ventricle. When the voltage threshold increased to 2 V and the impedance went from 450 to 800 ohms, six months postimplantation, Twiddler syndrome was not considered. Since the RV lead threshold and impedance stayed stable after the first elevation six months postimplantation, and pacing was not required, no chest X-ray was performed. However, a chest X-ray done at this time could have possibly established the diagnosis. The twisting of the electrode in the pouch might have damaged the electrode. The inability to place a stylet inside the electrode reveals the loss of integrity of the lead, which probably caused a progressive increase in impedance with conduction resistance.

In a cohort of 1074 patients who underwent cardiac implantable electronic devices, lead macrodislodgment occurred in 19 (1.8%) patients, and the vast majority (89%) of them were detected by a change in lead parameters on remote monitoring [6]. Hence, a remote monitoring system permits the early detection of Twiddler syndrome. Early diagnosis of defibrillation lead dislodgment typically involves a decrease in R-wave amplitude and impedance, with an increase in pacing threshold [7]. Twiddler syndrome should be considered when there is a simultaneous increase in pacing thresholds and lead impedance. Macrodislodgment is usually the manifestation in Twiddler syndrome, but in our patient, microdislodgment should be considered. The chest X-ray confirms a lead traction with loss of the curve. This traction might explain a reduced contact with the right ventricle and threshold elevation. Almost 50% of patients with Twiddler syndromes are asymptomatic or with unrelated symptoms [8]. In the case of lead parameter change detected in the clinic or with remote monitoring, even late after implantation, a chest X-ray should be performed to exclude a Twiddler syndrome.

Our patient was an elderly woman with an increased BMI who fulfilled three well-known risk factors for Twiddler syndrome: increased BMI, female gender, and elderly patient [6, 9]. Obesity limits the physical exam on the pocket site, which makes the assessment of Twiddler syndrome through the skin more difficult. Excessive loose subcutaneous tissue without fixation of the device in the pocket probably contributed to the mobility of the device in the pocket and allowed for rotation of the ICD spontaneously in daily life movements.

## 4. Conclusion

This case report highlights the utility of remote monitoring for early detection of lead abnormalities or arrhythmia, which can be caused by rare device complications such as Twiddler syndrome. Measures to identify risk factors for Twiddler syndrome and fixation of the device at time of implant in high-risk patients can prevent this unusual complication.

## Data Availability

The data used to support the findings of this study are included within the article.
